# Cannabidiol's neuroprotective properties and potential treatment of traumatic brain injuries

**DOI:** 10.3389/fneur.2023.1087011

**Published:** 2023-02-02

**Authors:** Mackenzie M. Aychman, David L. Goldman, Joshua S. Kaplan

**Affiliations:** Behavioral Neuroscience Program, Department of Psychology, Western Washington University, Bellingham, WA, United States

**Keywords:** cannabidiol, cannabis, traumatic brain injury, concussion, neuroprotection, neuroinflammation, marijuana, blood brain barrier

## Abstract

Cannabidiol (CBD) has numerous pharmacological targets that initiate anti-inflammatory, antioxidative, and antiepileptic properties. These neuroprotective benefits have generated interest in CBD's therapeutic potential against the secondary injury cascade from traumatic brain injury (TBI). There are currently no effective broad treatment strategies for combating the damaging mechanisms that follow the primary injury and lead to lasting neurological consequences or death. However, CBD's effects on different neurotransmitter systems, the blood brain barrier, oxidative stress mechanisms, and the inflammatory response provides mechanistic support for CBD's clinical utility in TBI. This review describes the cascades of damage caused by TBI and CBD's neuroprotective mechanisms to counter them. We also present challenges in the clinical treatment of TBI and discuss important future clinical research directions for integrating CBD in treatment protocols. The mechanistic evidence provided by pre-clinical research shows great potential for CBD as a much-needed improvement in the clinical treatment of TBI. Upcoming clinical trials sponsored by major professional sport leagues are the first attempts to test the efficacy of CBD in head injury treatment protocols and highlight the need for further clinical research.

## Introduction

Traumatic Brain Injury (TBI) is a global public health epidemic that causes death or hospitalization in an estimated 27–69 million people annually (1, 2). Yet, TBI has been called the “silent epidemic” because of its range in acute symptoms and severity that lead to underdiagnosis and underreporting by patients or treatment facilities (3–6). In addition to acute symptomology that includes amnesia, disorientation, and changes to mental processing speed, even mild TBIs can have long-term mental health impacts including depression and changes in impulsivity, judgement, and memory. The severity of the impact (i.e., the direct trauma to the brain) often determines the severity of the TBI symptoms (7) and involve brain changes that underlie persistent neurological deficits and seizures. These post-concussion symptoms contribute to high hospitalization rates among TBI sufferers in which 43% require additional hospitalization during the first year post-injury (5). Patients with TBIs have financial hardships caused by their cognitive and physical disabilities that can require expensive medical treatments and limit work activities. There is also the societal economic burden that in the United States, alone, was $76.5 billion in 2010 dollars (5). Because of inconsistent diagnoses and subsequent underreporting of TBIs, the true cost and financial impact is expected to be much higher than this estimate.

The complexity of cellular, molecular, physiological, and neurometabolic mechanisms associated with different stages post-TBI makes it particularly difficult to treat. There is currently no single pharmacological approach that has been effective in treating TBIs (8). Yet, shared mechanisms of damage exist across TBI severity levels suggesting that a single strategy may be generally efficacious (9). Research into Cannabidiol (CBD), a non-intoxicating phytocannabinoid abundantly produced by some chemovars of *Cannabis sativa L* or synthetically produced from several biological systems (10), has revealed promising protective properties to counter the damaging effects of TBI that warrant concentrated investigation (11–13). CBD's unique pharmacodynamic profile (14) and high tolerability in adults (15–17) affords unique capabilities not shared by currently available treatment strategies. Here, we discuss CBD's proposed protective mechanisms against TBI-induced neuroinflammation and degeneration, which may be a plausible intervention for treating and reducing physiological damage and the associated symptoms that arise from TBI.

## Mechanisms of injury

The clinical presentation of TBI symptoms results from a sequence of physiological, molecular, and chemical changes that occur immediately following the initial impact or following a delay. These two waves of disturbances are described as the primary and secondary injury, respectively.

### Primary injury

The primary injury of TBI is the direct structural damage to neural tissues and blood vessels that derive from the impact event itself (18). During impact, there is a shockwave of brain compression and expansion that creates substantial mechanical forces within the skull. This shockwave causes immediate contusions to the area of impact, damages glial cells, induces localized hemorrhaging, shears axons and blood vessels, and disrupts cytoskeletal elements (19–23). Currently, helmets and mouthguards are the only known protective strategies against primary TBI injury resulting from non-vehicular sport or combat activities. These protective devices are effective at reducing the severity of TBIs but cannot prevent TBI when there is sufficiently forceful impact. Thus, a variety of TBIs occur both with (e.g., military combat) and without this protection (e.g., motor vehicle crashes). Due to the nature of the immediate physiological damage, pharmacological approaches are intended to reduce secondary injury rather than prevent the occurrence of primary injury.

### Secondary injury

The secondary injury is the additional systems damage that results from mechanisms induced by the original structural injury (i.e., the primary injury). It is characterized by a widespread cascade of cellular, molecular, and biochemical changes that include, but is not limited to, unregulated ion and neurotransmitter release, dysregulation of glial cells, neuronal hyperexcitability, excitotoxicity, increased blood brain barrier (BBB) permeability, and widespread neuroinflammation (24–27). Diffuse mechanoporation results in ion leakage and ultimately the unregulated release of glutamate that triggers apoptotic events (24, 28, 29), increases the presence of reactive oxygen species (ROS), and induces oxidative stress (30). Together, these outcomes further trigger a proinflammatory response involving microglial and macrophage activation and pro-inflammatory cytokine release that boost and sustains posttraumatic inflammation for an indeterminant amount of time (31). This protracted inflammatory response contributes to additional tissue damage and neurodegeneration (32, 33). Activated microglia in their M1 state are especially neurotoxic because of their ability to enhance pro-inflammatory and neurotoxic mediators [e.g., IL-1β, tumor necrosis factor (TNF)-α, superoxide radicals, nitric oxide] and decrease phagocytic activity (34). The resulting immune response and concurrent neuroinflammation create a toxic cellular environment that inhibits neuronal healing and perpetuates neuron loss (35) and atrophy (36), and is associated with white matter degradation (37). Therefore, this inflammatory cascade is a main target for pharmaceutical intervention (26).

## Current clinical treatment practices and the role for CBD

**Figure 1:**
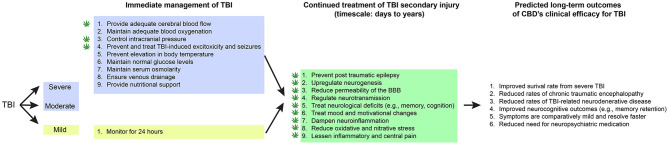
CBD's proposed role in immediate and continued treatment of TBI symptoms. TBI severity determines the scope of immediate clinical interventions. Preclinical evidence supports CBD's potential utility in some of these immediate treatment procedures (indicated by a cannabis leaf). However, CBD has broader potential to support TBI recovery by dampening the secondary injury cascade. If CBD is effective at improving some of these symptoms, there would be long-term predicted benefits across survival, neurocognitive, neurodegenerative, and neuropsychiatric measures.

Physicians managing patients with TBI are seeking safe and effective treatment options that would ameliorate the symptoms of TBI, reduce recovery time, and prevent or decrease chronic neurologic dysfunction from residual brain injuries (Figure 1). Similar underlying mechanisms of secondary injury are observed across TBI severities, but differences in their magnitude impact clinical treatment strategies and patient outcomes (38). To clinically verify that pharmacotherapy such as CBD would improve outcomes for patients with mild, moderate, and severe TBI, there must be a reliable way to determine the severity categories.

There is ongoing debate in the medical community about the reliability of current clinical tools, such as the Glasgow Coma Scale (GCS), to assess and categorize TBI severity (39). The GCS measures the level of consciousness and has been commonly used to classify TBI severity as mild (GCS 13–15), moderate (GCS 9–12), and severe (GCS 3–8). Numerous influential medical organizations, including the World Health Organization, classify TBI using this GCS scale (40–42). However, one-third of trauma patients with GCS score of 13 were found to have intracranial lesions (43) raising concerns that these patients should not be categorized as mild TBI and instead categorized as experiencing moderate severity. Subsequently, recommendations were made to redefine mild TBI (GCS 14–15) and moderate TBI (GCS 9–13) (44). There are also ongoing discussions of ways to improve current TBI assessment and develop new tools that facilitate both clinical research and treatment (39).

One approach to improve the assessment of TBI severity is to incorporate more clinical information, exemplified by the work of the American Congress of Rehabilitation Medicine (ACRM). In 1993, ACRM defined mild TBI as an event causing one or more of the following conditions: (1) loss of consciousness (LOC), (2) post traumatic amnesia (PTA), (3) mental status changes, and (4) focal neurologic deficits. The severity of this TBI was assessed by how long the patient was unconscious, how long the PTA lasted, and the GCS score [see Brasure et al. (45) for specific criteria]. Notably, the ACRM's original definition only pertained to mild TBI but the same measures of severity have been widely used to categorize mild, moderate, and severe TBI (41). In 2021, the ACRM announced plans to update their work on measures of TBI severity to more reliably distinguish a mild TBI, from moderate and severe injuries (46). Being able to match CBD's outcomes to reliably assessed TBI severities will lead to better predictive validity and more effective long-term care plans.

### Clinical management of different TBI severities

It is estimated that mild TBI represents 70–90 percent of all traumatic brain injuries (47, 48). Even mild TBI can have symptoms that are severe and debilitating such as posttraumatic headaches, nausea/emesis, dizziness or impaired balance, light or noise sensitivity, blurry or double vision, fatigue, memory impairment, poor concentration, increased anxiety, irritability, emotional lability, worsened mood, and sleep disturbance (49). Patients suspected of having a mild TBI should be medically evaluated by a licensed health professional to provide clinical management, head injury instructions, and determine whether they will need referral to an emergency department for a head CT to evaluate for more dangerous brain injuries that require hospitalization (50).

Current management for mild TBI recommends observation for 24 h at a hospital or home to watch for neurologic deterioration (50). These patients are also instructed to refrain from strenuous activities and contact sports until the symptoms resolve which usually occurs around 4 weeks (51) with notable variability (52). Clinical research is needed to determine whether CBD can stop or reduce the severity of any of the symptoms caused by mild TBI and whether treatment with CBD reduces the recovery time. Another clinical issue is whether treatment with CBD lowers the risk of permanent injuries from one or multiple episodes of mild TBI and lowers the risk of CTE associated with multiple head injuries.

Patients with moderate and severe TBI can have primary injuries that require surgical intervention including: depressed skull fracture, subdural or epidural hematoma, intracerebral hemorrhage, and penetrating injury (44, 53). Hospitalized patients with moderate and severe TBI also require medical management, usually in the intensive care unit, with the goal of controlling secondary injuries (44, 53).

Current treatment of intermediate and severe TBI in a critical care unit (ICU) includes medical and surgical management known to mitigate the effects of secondary brain injury: (1) maintaining blood pressure to provide adequate cerebral blood flow, (2) maintaining adequate oxygenation and normal pCO_2_, (3) controlling intracranial pressure (ICP), (4) preventing and treating seizures, (5) preventing elevated body temperature, (6) maintaining normal glucose levels, (7) management of IV fluids to maintain normal serum osmolarity, (8) elevating and positioning the head to promote venous drainage, and (9) provide nutritional support (44, 53). The patient is at considerable risk of deteriorating to brain death if there is a progression of cerebral edema and elevated ICP that cannot be controlled. Bifrontal craniectomies with dural incisions to decompress the brain is a surgical option for patients not responding to medical management of elevated ICP. This is a lifesaving procedure that effectively reduces ICP but usually leaves survivors with severe disabilities (54). Despite the slow recovery, and prolonged rehabilitation, some of these patients regain enough activities of daily living to provide a degree of functional independence (53).

### A clinical role for CBD

The clinical issue is whether CBD, a treatment that can decrease BBB permeability and reduce neuroinflammation (55), can be an effective tool to reduce cerebral edema and lower ICP, and reduce the number of patients who develop this life-threatening intracranial hypertension. CBD differs from current clinical management options because it directly repairs the underlying cause of vasogenic edema which include increased permeability of the BBB (56) and neuroinflammation (57). As presented later in this review, pre-clinical research has demonstrated CBD's efficacy against these TBI consequences as well as improve cerebral blood flow (58) and treatment of genetic and pharmacologically-induced seizures (59). However, clinical research is needed to verify that CBD can be an effective pharmaceutical agent for reducing cerebral edema in patients with moderate and severe TBI. This clinical research also needs to evaluate whether CBD can reduce the incidence of post traumatic epilepsy (PTE), and if improved management of CBF contributes to a better clinical outcome. There is also preclinical evidence that CBD promotes neurogenesis (60), and the clinical issue is whether the neurogenesis that normally occurs in TBI patients can be enhanced with CBD and if this will reduce head injury symptoms, speed the recovery, and provide a better restoration of neurological function. Following in-patient treatment and stabilization period, patients may be released with substantial impairments in brain function that results from their TBI. This damage to neurotransmitter signaling results in long-lasting changes in personality traits, mood, and neurological function. Below, we describe these changes and the relevant mechanisms through which CBD may target them.

## Damage to neurotransmitter systems

Both primary and secondary injury cascades impact neurotransmitter systems that result in sustained imbalance of cortical excitation and inhibition (24). Rodent models of TBI have implicated shifts in glutamate, GABA, serotonin, and catecholamine signaling in the persistent disruptions and damage to neural circuits (25).

Compression of tissue during TBI causes morphological damage and leads to a rapid increase in intracellular Ca^2+^ (61–63). This elevation in intracellular calcium contributes to the excessive glutamate release and region-specific changes in excitatory amino acid transporter (EAAT) expression that's observed following TBI (64–66). The outcome is a reduction in glutamate uptake resulting in excitotoxicity and apoptosis (67, 68). In response to elevated synaptic glutamate concentrations, AMPA receptors undergo shifts in subunit expression and become calcium permeable (69) which may enhance plasticity following injury, but an ultimate reduction in NMDA signaling—following a robust increase post-injury—reduces excitatory output of affected neurons (70, 71). Together, the initial wave of acute posttraumatic glutamate release results in excitotoxicity, apoptosis, and dysfunction of surviving neurons, while the following depression of signaling is responsible for some motor and cognitive deficits associated with TBI (24).

In parallel, the loss of GABAergic neurons elevates the disparity in the excitation/inhibition ratio and augments apoptotic processes and cellular injury. Apoptotic events resulting from the primary injury and an increase in extracellular glutamate promote the loss of GABAergic neurons. Furthermore, TBI induced dysfunction to GABA_A_ receptor subunits (decreased α1, α4, γ2, and δ) leads to abnormal patterns of phasic and tonic inhibition, with a higher reduction in phasic inhibition (72). Additionally, activation of the JaK/STAT and Egr3 pathways results in decreased GABA_A_ receptor signaling and hyperexcitability (72–75). This GABAergic neuron loss and reduced inhibitory tone is associated with both cognitive and motor deficits in addition to TBI-induced seizures (74). The remaining GABA neurons show elevated levels of GABA_B_ receptor signaling (76), which serve as autoreceptors and further decrease GABA_A_ receptor-mediated transmission. Changes to the expression of genes regulating glutamate and GABA result in long-lasting changes in homeostatic control and hinder endogenous mechanisms to restore the excitatory/inhibitory balance (25).

TBI negatively impacts attention, memory, and mood through impairments to cholinergic (77) and serotonergic systems (78). Biphasic changes in acetylcholine signaling begin with initial unregulated acetylcholine release and are followed by chronic cholinergic hypofunction (77, 79). This is eventually reflected in decreased receptor binding (80) and reduced choline acetyltransferase activity (81). Ultimately, this results in impaired attention and disrupted memory consolidation (82). Mood changes following TBI are thought to stem from sustained decreases in both serotonin receptors and transporters, contributing to increased rates of anxiety and depression in TBI sufferers (83).

TBI also triggers an increase in tyrosine hydroxylase and enhances the synthesis of dopamine and norepinephrine (84–86). However, persistent neuroinflammation contributes to a downregulation of receptors and decreased transmitter release (87, 88). Further, direct axonal damage from the primary injury negatively impacts signaling (89, 90). Sustained dopamine impairment can have feed-forward effects that further increase inflammation (91), disrupt metabolism (92), and reduce levels of brain-derived neurotropic factor (BDNF) (93), which normally has a role in stimulating neurogenesis, promoting neuronal survival, facilitating regeneration, and protecting tissue from oxidative stress and apoptosis following TBI (94). Lower baseline levels of BDNF among older adults hinders recovery and is one factor that may lead to lower rates of survival from TBI (95).

The consequence of TBI on several neurotransmitters systems causes many of the clinical and pathological hallmark symptoms of injury and underlie changes to cognitive and motor processes. Furthermore, structural damage to the lesioned areas contributes to mood and cognitive disturbances, such as damage to the forebrain cholinergic (96, 97) and catecholaminergic afferents (86, 98). An effective pharmacological approach will protect against any perturbation to these systems to prevent further neurological damage from the secondary injury response.

## The impact of ECS signaling on neuroinflammation during secondary injury

The endocannabinoid system (ECS) influences TBI outcomes as increasing ECS tone protects against synaptic hyperexcitability, reduces neuroinflammation, and improves blood brain barrier integrity (99–101). The ECS is comprised of cannabinoid type I and II receptors (CB_1_ and CB_2_) and lipid signaling messengers, anandamide, and 2-AG. Among its numerous functional roles, its powerful influence over the immune response and neuroinflammatory signaling confers neuroprotective qualities that are relevant in the TBI secondary injury cascade. Endocannabinoids are involved in regulating inflammation by acting on cannabinoid receptors as well as targets beyond the canonical endocannabinoid system including TRPV1 and PPARγ (102). Yet, their neuroprotective potential has been most thoroughly studied at the endocannabinoid receptors. CB_1_ receptors are expressed in a variety of cell types and tissues but are expressed in highest abundance in the central nervous system (CNS) (103). CB_1_ receptors are involved in regulating inflammation in both the CNS and PNS and play an important role in dampening proinflammatory chemokine secretion (104). CB_1_ activation inhibits adenylate cyclase activity and decreases levels of cAMP, in addition to activating inwardly rectifying K^+^ channel conductance, decreasing N-type and P/Q-type voltage-operated Ca^2+^ channel conductance, ultimately reducing intracellular Ca^2+^ influx (105). The consequence of CB_1_ activation is therefore a reduction in neurotransmitter release, however its net impact on glutamate vs. GABA release is regionally determined and based on endocannabinoid concentrations (106–108).

CB_2_ receptors are present in higher concentrations in the immune system and on microglia than on neurons (55, 103, 109, 110). They have notable immunomodulatory and neuroprotective roles *via* the MAPK pathway and regulation of ERK-_1/2_ phosphorylation, which aids in reducing inflammation (109). Additionally, activation of CB_2_Rs is important for decreasing M1 state macrophages and increasing bias toward anti-inflammatory M2 polarization (111). CB_1_ and CB_2_ are highly involved in TBI and a potential reduction in inflammation and Ca^2+^ influx, which can prevent excitotoxicity, inhibit inflammatory cytokine production, and may therefore be neuroprotective (105).

Previous studies in a mouse model of TBI have demonstrated that CB_1_ and CB_2_ antagonists prevent activation of neuroprotective mechanisms in response to brain edema, diffuse axonal injury, and microglial activation (101, 102, 112). Therefore, endogenous ECS signaling can be neuroprotective, but it can also be a target of therapeutic intervention. For instance, boosting 2-AG levels protects against neurodegeneration, normalizes ionotropic glutamate and GABA_A_ receptor expression levels, prevents additional tau pathologies, and improves behavioral outcomes in a mouse model of repeated TBI (113, 114). Since CB_1_ and CB_2_ antagonists only partially blocked neuroprotective benefits of increasing 2-AG levels, it suggests that ECS neurotransmitters may confer neuroprotection against secondary injury through actions at both ECS and non-ECS receptors. One potential non-ECS receptor target could be δ-subunit containing GABA_A_ receptors (115) that are found extrasynaptically and mediate a tonic-inhibitory current (116). Further mechanistic investigation is needed to determine the ECS and non-ECS receptor contributions to neuroprotection in TBI. Importantly, the neuroprotective potential of solely targeting the ECS receptors is limited by the sensitivity to desensitization following prolonged pharmacological activation (117, 118). Therefore, pharmacological interventions for TBIs may be more effective if they target additional mechanisms beyond solely CB_1_ and CB_2_ receptors.

## CBD's neuroprotective potential in TBI

Cannabidiol (CBD) is a non-intoxicating, non-psychedelic phytocannabinoid that has over 65 known ECS and non-ECS targets in the brain and body that are influenced dose-dependently [for a detailed review of relevant molecular targets, see (14)]. Not all of these targets are relevant in the context of TBI therapy, but several of these targets directly mitigate inflammation which has prompted interest in the potential utility of CBD to dampen secondary injury in TBI. These relevant targets and therapeutic mechanisms are discussed below. However, there are currently no clear dosing guidelines for these neuroprotective benefits in TBI. Effective CBD dosing has proven challenging for other therapeutic purposes such as anxiety (119), deficits in prosocial behavior (120), and cocaine-induced reinstatement (121) that demonstrate inverted-U dose response curves. It's unclear if a similar inverted-U response will be observed for CBD's neuroprotection against the multitude of secondary injury cascade mechanisms or if treatment efficacy is retained with escalating dosing such is observed with CBD's anti-epileptic effects (120, 122).

**Figure 2:**
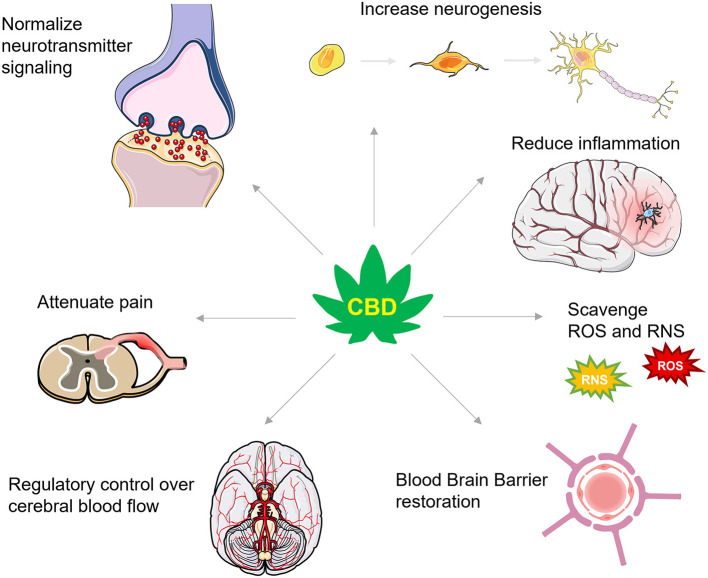
A summary of CBD's actions in TBI. CBD has numerous actions that are proposed to protect against secondary injury and support recovery from TBI. These actions include effects on numerous neurotransmitter systems that increase levels of brain derived neurotrophic factor and enhance neurogenesis, dampen inflammatory signaling cascades, scavenge for reactive oxygen and nitrogen species (ROS and RNS, respectively), restore the integrity of the blood brain barrier, improve control over cerebral blood flow, and attenuate inflammatory and neuropathic pain.

Achieving the optimal therapeutic dose for combating secondary injury in TBI will likely depend on CBD's bioavailability (123) which varies as a function of consumption method [for a detailed review of CBD's pharmacokinetics in humans, see Millar et al. (123)]. Pulmonary, sublingual, and intranasal absorption of CBD promote the highest level of bioavailability, but these administration routes have not been systematically assessed for combating the secondary injury cascade. Nonetheless, as we discuss below, CBD's neuroprotective efficacy during secondary injury has been demonstrated in several preclinical models accompanying restoration of TBI-impaired molecular, chemical, and physiological mechanisms that would be theoretically predictive of an effective TBI pharmacological strategy in humans. These mechanisms include, but are not limited to, increasing ECS signaling and reducing glutamate excitotoxicity, promoting neurogenesis, dampening neuroinflammation, scavenging reactive oxygen species, reducing TBI-induced BBB permeability, and regulating cerebral blood flow, all discussed in further detail below (Figure 2). Alleviating these consequences is beneficial for protecting against cognitive, mood, and motor changes and helping to better restore function following TBI.

One important consideration regarding CBD's neuroprotective efficacy is whether CBD needs to have reached the brain prior to the primary injury or if it can be effective if administered during the secondary injury response. Addressing the time course of CBD's efficacy will be a critical experimental consideration moving forward. There is currently insufficient evidence to draw any definitive conclusions because in many cases, CBD is administered prior to the primary injury [e.g., (12)], after [e.g., (13)], or both before and after [e.g., (56)], and often not compared across administration period. Pretreatment may lead to lower mortality rates and improved sensorimotor function (12), but targeting at least some of CBD's mechanisms, like the ECS, within a “window of opportunity” of at least 15 min following the primary injury may also confer substantial neuroprotective benefits (124). Given that the window of opportunity for CBD administration is not clearly defined or consistently tested, we discuss the following neuroprotective effects across a range of administration methods and temporal relationships to the primary injury.

### CBD's effects on neurotransmitter systems after TBI

CBD's neuroprotective effects likely stem from action at both ECS and non-ECS targets. CBD indirectly activates CB_1_ and CB_2_ receptors by acting as a competitive substrate for the FAAH enzyme that's responsible for the primary hydrolysis of the endocannabinoid anandamide (125). CBD may also increase 2-AG, but this is debated and may only occur regionally such as in the periaqueductal gray (126, 127). CBD may have some action directly on the ECS receptors, themselves. *In vitro* assessments suggest that CBD has low binding affinity for CB_1_ receptors and can act as a weak positive allosteric modulator of CB_2_ receptors (128). Together, CBD can stimulate ECS receptors by elevating anandamide levels and potentially by directly acting upon them to reduce neuroinflammation during secondary injury processes (111, 129, 130).

Pre-clinical models suggest that CBD may normalize the glutamatergic and GABAergic signaling imbalance following TBI. CBD administered prior to TBI reduced the increase in cortical glutamate release (12). This benefit was maintained with continued CBD administration for at least 30 days post-TBI. In this case, CBD's effects were observed at moderate (50 mg/kg) and high (100 mg/kg, 200 mg/kg) oral doses, but the 100 mg/kg dose was optimal suggesting that CBD's ability to restore normal glutamate levels may follow a similar inverted U dose-response curve to other conditions [e.g., (110)], albeit one with a much higher dose tolerance. Additionally, CBD blocks glutamate toxicity in cultured cortical neurons independent of inhibiting AMPA, NMDA, or kainite receptors (131). More direct dampening of glutamatergic excitotoxicity may occur through CBD stimulation of adenosine A2a signaling and resulting reduction in glutamate release (132–134). CBD's multi-target pharmacological mechanism may end up being safer and more efficacious than glutamate receptor antagonists which are insufficient for preventing neuronal dysfunction and resulting clinical symptoms (135), and may have adverse consequences such as increased risk for tumor growth (136). In addition to dampening excessive glutamate signaling, CBD increases GABAergic signaling *via* positive allosteric modulation of non-benzodiazepine binding sites on α-containing GABA_A_Rs, with preference for the α2-containing receptor subtype and the β2 or β3 subunit (137). Together, CBD effects restoration of the excitatory/inhibitory balance through direct and indirect actions on GABAergic and glutamatergic signaling, which may promote improved cognitive and pain symptoms that result from TBI.

Many of the alterations in mood following TBI may stem from impaired dopaminergic and serotonergic signaling (90, 138–140). The primary TBI injury can cause mechanical damage and shearing to dopaminergic axonal projections, which can additionally cause oxidative stress (90). Sustained damage to this neurotransmitter system can impair dopamine synthesis and metabolism (86). CBD aids in the neuroprotection of dopaminergic pathways, as it is a partial agonist at D2 and D3 receptors (141, 142) in rat striatal tissue, and reduces dopamine uptake (14, 143), which aids in attenuating the loss of dopaminergic neurons and microglial activation (134). The neuroprotective properties of CBD further aid in protecting the remaining neurons, dendrites, and cellular structures in dopaminergic pathways (144, 145).

Furthermore, CBD is a direct agonist of 5HT_1A_ receptors and a partial agonist on 5HT_2A_ receptors (14, 146–148), decreasing anxiety, pain and headaches associated with TBIs *via* interaction with the orthosteric binding site (141, 149–151). *In vitro* studies demonstrate that CBD 5HT_1A_R binding increases GTP binding to the 5HT_1A_R coupled G protein, Gi, confirming its role as an agonist. However, it should be noted that 5HT_2A_R agonism is only applicable in the presence of high concentrations of CBD (147). *Via* this action, CBD increases serotonergic and glutamate cortical signaling, as well as inhibits adenosine uptake (152). Rebalancing these neurotransmitter systems is important for TBI recovery, as reducing serotonin and adenosine reuptake results in decreased oxidative stress, excitotoxicity, and inflammation, which are hallmarks of TBI secondary injury and the main focus of pharmacological intervention (153, 154). SSRIs have been shown to improve common depressive symptoms post-TBI, which arise partly due to decreased serotonergic signaling (153). Increasing extracellular serotonin after damage has been shown as beneficial for improving mood disturbances, however it is ineffective in improving cognition (155). Therefore, it is important to use a pharmacological approach that addresses both mood and cognitive symptoms that arise from TBI.

CBD also antagonizes the G protein-coupled receptor GPR55 (156), which results in enhanced GABAergic neurotransmission in the mouse hippocampus due to increases in inhibitory neuron excitability, and ultimately decreases the excitation/inhibition ratio (120). Inhibiting GPR55 reduces Ca^2+^ release, and therefore aids in further reduction of glutamate release and excitotoxicity (14).

Together, CBD has widespread effects attributed to interactions with non-ECS targets, such as PPARγ (157), 5-hydroxytryptamine (5-HT_1A_) receptors (158), adenosine receptors (159, 160), vanilloid receptor 1 (TRPV1) (161, 162), G-protein coupled receptors (i.e., GPR18 and GPR55) (120, 141, 156, 163), GABA_A_ receptors (137), and glutamate receptors (131, 164). CBD's effects on these various neurotransmitter mechanisms and the restoration of the excitatory/inhibitory balance are proposed to improve mood and pain-related symptoms of TBI, and limit long-term damage caused by excitotoxicity and inflammation. Notably, the net consequence of CBD's actions on these different pharmacological targets will need to be evaluated in humans with TBI since research into CBD's effect on the brain of adults with autism spectrum disorder show differential effects as a function of disease state and pathological etiology (165, 166).

### CBD's effect on neurogenesis

As part of the natural healing process following TBI, neurogenesis is increased in the hippocampus, cerebral cortex, and white matter in rodents (167, 168). Additionally, neural stem/progenitor cell protein markers naturally increase in humans post-TBI, indicating that neurogenesis may be induced in humans in the same way as in an animal TBI model (167). This generation of new cells aids in cognitive and spatial TBI recovery (169). CBD can further promote neurogenesis through multiple mechanisms such as increasing CB_1_ (170) or PPARγ receptor signaling (171) to regulate stem cell proliferation and differentiation (172) in the granule cell layer of the hippocampus. Therefore, CBD's actions on ECS and non-ECS mechanisms may regulate neurogenesis (173), but they may do so in a dose-dependent and condition-dependent manner. In unstressed animals, CBD's pro-neurogenic effects appear to be dose-dependent, with lower doses promoting neurogenesis and higher doses suppressing it (174). The impairment to neurogenesis at high doses was postulated to result from desensitization of CB_1_ receptors. However, in chronically-stressed animals, higher doses retained pro-neurogenic effects (60). Therefore, the dose-dependency of CBD's effects on neurogenesis may depend on symptom etiology where different CBD targets mediate its effects. At this time, it is unknown how CBD's targets and dose-dependent mechanisms are affected by TBI across the range of severities. Further work is needed to clarify the dose-dependency of CBD on neurogenesis in TBI and identify whether these benefits also exist from CBD treatment in humans. Even though plasma CBD concentration was positively associated with hippocampal volume in heavy cannabis users (175), its direct impact on human neurogenesis remains undetermined.

Together, CBD's actions on formal ECS and related targets counteract the pro-inflammatory cascade and may enhance the neurogenic response to TBI. Although CBD is often considered in light of its effects on ECS signaling, its numerous non-ECS targets likely contributes to the range of effects it can have on secondary injury in TBI.

### CBD and inflammation

Microglia play an important neuroprotective role in the healthy brain by serving as the first responders to injury or disease (176). They are responsible for clearing cellular waste and plaques and aid in neutralizing infectious agents. However, their chronic activation can damage healthy cells and is thought to contribute to numerous neuropathologies including the damage that occurs during the secondary injury cascade of TBI. Dampening microglia activation following TBI may be a targetable mechanism for limiting the extent of secondary injury damage.

Primary TBI damage ruptures axons and their myelin sheaths releasing ATP that binds to damage-associated molecular pattern (DAMP) receptors on microglia and activates their M1 pro-inflammatory state (28, 177). This proportion of polarized microglia in their M1 state is positively correlated with the severity of white matter injury (177). Microglia in their M1 state release toxic substances such as inflammatory cytokines that are important for neuroprotection from minor injury or infection (34, 178–180). Microglia that persist in this M1 state can trigger excessive and pathological synapse degradation and can induce neurotoxicity due to the release of neurotoxic mediators and pro-inflammatory factors, which creates cycles of microglial-mediated neurodegeneration (34, 180–182).

One of CBD's proposed protective mechanisms involves biasing the polarization of these microglia from its damage-causing pro-inflammatory (M1) to the anti-inflammatory (M2) activation state (111, 183). This action is mediated through indirect action at A_2A_ adenosine receptors or by enhancing AEA-mediated CB_2_ receptor signaling (133, 184, 185). CBD's ability to shift the active state of microglia is an important protective mechanism against the ongoing damage to synaptic integrity that occurs during the secondary injury cascade.

### CBD and oxidative/nitrative stress

TBI increases free radical synthesis at a rate higher than can be suppressed by the endogenous scavenger system resulting in oxidative stress (186, 187). The accumulation of these free radicals during secondary injury damages nearby cells and contributes to cellular damage as they attempt to supplement their missing electron through oxidation or harmful scavenging of electrons from nearby proteins, membrane, and DNA. Activated microglia in their M1 state are a primary source of these free radicals as they increase reactive oxygen species and reactive nitrogen species as part of the proinflammatory response to neural injury (188).

One facet of CBD's neuroprotective mechanisms may stem from its potent antioxidant and antinitrative effects in addition to its indirect ability to dampen ROS generation (189, 190). CBD's molecular structure confers strong antioxidant properties; it has electrophilic-hydroxyl groups in its aromatic phenol ring which allows it to be readily oxidized (131, 189, 191). It can also prevent the formation of superoxide radicals and ROS through numerous mechanisms including blocking oxidase activity, chelating transition metal ions (192), and affecting the levels and activity of antioxidants (189). CBD can further affect the redox balance by elevating AEA levels and reducing oxidative stress through a CB_2_-dependent mechanism (193, 194). Together, CBD's antioxidant properties serve as an additional neuroprotective mechanism that protects cellular function, stability, and prevents the initiation of apoptotic cascades (192).

### CBD and BDNF

BDNF is an important neurotrophin involved in the recovery process from TBI. Serum BDNF levels on the day-of-injury for adults with non-severe forms of TBIs can be used as a prognostic indicator, as high levels predict better recovery (195, 196). BDNF is a secreted autocrine factor that promotes the regeneration, synaptogenesis, axonal sprouting, and survival of neurons (195, 197, 198), which are critical factors in effective neurogenesis. It is involved in reducing the secondary cascade of injury in TBI by restoring functional connectivity and providing further neuroprotection (199, 200). BDNF is found in higher concentrations in the cortex and hippocampus immediately post-TBI but decreases in concentration within the first 24 h due to a molecular cascade involving phosphorylation of PERK (a protein-kinase in the endoplasmic reticulum activated by stress) and increased activation of CREB, which downregulates BDNF (201). Impairments in dopamine signaling are partly responsible for the sustained reduction in BDNF levels that follow and contributes to memory, cognitive, and depressive symptoms (55, 94).

Boosting BDNF levels may have utility in combating secondary injury. In animal models, a positive correlation has been shown between increased BDNF expression and improved functional outcomes in terms of motor, memory, behavior, and cognitive responses after TBI. A decrease in BDNF levels during secondary injury is associated with worse outcomes (195). In human studies, acute serum BDNF levels are associated with chronic memory impairments, functional cognitive limitations, and depressive symptom severity, with an inverse correlation between BDNF levels and cognitive impairment (95, 202, 203). These beneficial effects of BDNF are likely mediated through TrkB receptors, resulting in intracellular signaling cascades that enhance relevant protein synthesis (202).

CBD's ability to increase BDNF levels proposes an additional neuroprotective mechanism against secondary injury. CBD has been shown to increase synthesis of BDNF in the prefrontal cortex and hippocampus in mice (202), two regions implicated in the cognitive, memory, and mood-related impairments following TBI (204–206). However, it's unclear if CBD can rescue cognitive deficits and mood changes following TBI if these changes are associated with tissue damage and not merely altered neurotransmitter signaling (204, 205). CBD may be more effective in rescuing mood and memory deficits caused by shifts in hippocampal signaling from TBI-induced volume reduction (206) or increased amygdala activity (207). For instance, the increase in BDNF levels by CBD protected against neurotransmitter alterations and coinciding depression-like symptoms in a mouse model of brain ischemia (208). However, this neuroprotective mechanism remains speculative since there are currently no known studies to have directly assessed the role of CBD-induced upregulation of BDNF in TBI and whether this neuroprotection extends to TBI with severe tissue damage from the primary injury.

### CBD and the blood brain barrier

The BBB is a specialization of the blood vessels that vascularize the CNS. It regulates the movement of ions, molecules, and cells between the blood and the brain to prevent the brain's exposure to neurotoxins and infectious agents. Tight junction networks that are established by capillary endothelial cells in the CNS protect neural tissue from bacteria, viruses, toxins, and pathogens (209). Disruption increases toxin exposure, elevates immune and inflammation responses, and disturbs the brain's biochemical environment (56).

Both the primary TBI and secondary TBI cascade enhance the BBB's permeability. The shearing force of the primary injury can cause injury to endothelial cells and a loss of blood flow resulting in decreased BBB integrity that enables the passage of potential toxins into the CNS (209). This breakdown triggers leukocyte recruitment that promotes the release of proinflammatory cytokines or ROS, activates M1 microglia, and increases neuroinflammation and apoptosis (210–214). During the secondary injury cascade, increases in oxidative stress, transforming growth factor beta 1 (TGF-β1), and TNF-α promote a further reduction in the integrity of the BBB through suppressing the tight-junction protein, claudin-5 (215, 216). This leads to protracted hyperpermeability of the BBB and neuroinflammation.

**Figure 3:**
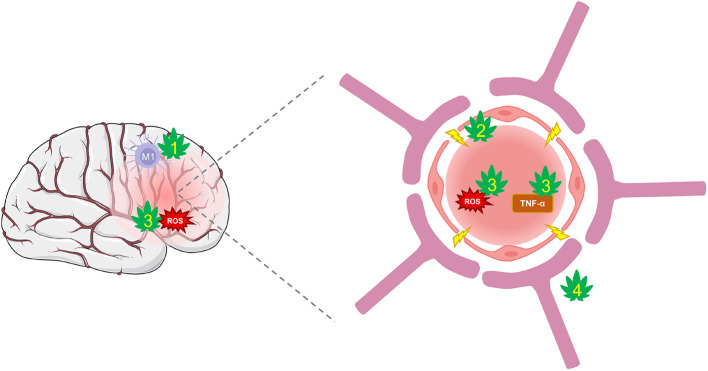
CBD protection against damage from BBB disruption. TBI disrupts cerebral blood flow and damages the integrity of the BBB. Hyperpermeability resulting from damaged tight-junctions and endothelial cells leads to increased inflammation and oxidative stress. (1) CBD shifts the polarization of macrophages from their pro-inflammatory M1 type to anti-inflammatory M2 type *via* activation of A_2A_ adenosine receptors or by enhancing AEA-mediated CB_2_ receptor signaling. (2) CBD may improve BBB integrity and prevent hyperpermeability by suppressing TBI's damaging effects on tight-junction proteins *via* action on PPARγ and 5-HT_1A_ receptors. (3) CBD is a potent antioxidant that reduces ROS and protects against oxidative damage to neurons and the BBB. It also reduces levels of TNF-α and other inflammatory markers that reduce the integrity of the BBB. (4) CBD may regulate cerebral blood flow to enhance reperfusion following injury *via* activation of GPR18, GPR55, and 5-HT_1A_ receptors.

Pre-clinical studies have exposed CBD's potential to suppress BBB permeability through several protective mechanisms (Figure 3). CBD can protect against the suppression of tight-junction proteins, claudin-5 and occludin (56). These actions may be mediated, in part, through PPARγ and 5-HT_1A_ receptors that have been shown to maintain function (217). Further, CBD decreases TNF-α which prevents further BBB damage and limits the inflammatory response (152, 218). Additional CBD targets, such as TRPV1, GPR18, and 5-HT_1A_ are involved in regulating cerebral blood flow and conferring further protection against cerebrovascular impairments from TBI (58, 219–221). The combinatorial effects of CBD acting on numerous targets may be a promising strategy of protecting the BBB's integrity and limiting feed-forward damage that occurs during secondary injury.

### CBD and cerebral blood flow

Adequate and controlled cerebral blood flow (CBF) is essential to neurological function and is an important clinical consideration following TBI (222). Various clinical studies have demonstrated an acute transient decrease in CBF following TBI, which returns to baseline during the recovery process (223–225). Large reductions in CBF are associated with worse neurological outcomes post-injury (226, 227), while acutely increasing CBF following TBI aids recovery (228).

A variety of local chemical mediators of CBF are altered after TBI, including an increased release of K^+^ into the extracellular space, excessive release of excitatory amino acids, increased acidity of cerebral pH, and increased interstitial adenosine. The resulting impairment in CBF autoregulation causes endothelial dysfunction and vasospasm while boosting release of free radicals (229–233). Disturbances to the autoregulatory mechanisms may increase vulnerability to additional secondary injury cascades and further dysregulate CBF and sensitivity to hypotension (222).

CBD action on GPCR18 (an endothelial cannabinoid receptor) and GPR55 may aid in recovering regulatory control over CBF *via* vasomotor control (55, 234). CBD administration to mice before and after an experimental middle cerebral artery occlusion protected against CBF impairment *via* action on 5-HT_1A_ receptors, thereby illustrating CBD's potential efficacy to enhance reperfusion following injury (235, 236). Therefore, there's emerging and supporting pre-clinical evidence that CBD may act as a cerebroprotectant by protecting against the dysregulated CBF from occlusion or injury.

### CBD and pain

Given the physical trauma of the primary TBI, inflammation-induced nociception is usually a primary symptom of injury. CBD acts both at the site of injury (see discussion above) and centrally to achieve its pain-relieving effects in pre-clinical models. For instance, microinjection of CBD directly into the nucleus accumbens attenuated pain responses during both the early and late phases of the formalin test but were strongest in the late phase (237). Since plasticity in the dorsal horn generated from excessive C-fiber input is believed to contribute to late phase nociception (238), these findings suggest that CBD may dampen inflammatory and neuropathic pain. Others have demonstrated the importance of brain 5-HT_1A_ (239) and D_1_ and D_2_ dopamine receptors (240, 241), as well as TRPV1 receptors in the spinal cord (162) in promoting these centrally mediated pain-reducing effects. Nonetheless, CBD's efficacy as a pain treatment in humans may vary due to route of consumption, dose, or pain etiology. At this time, there's no empirical evidence that CBD is effective at treating posttraumatic headache. However, CBD's ability to regulate inflammation may help prevent the sensitization of the trigeminal pain circuit (242) that underlies posttraumatic headache (243). Further research is warranted for specifically investigating CBD's potential as a pain treatment from TBI etiology.

## Discussion

### Current limitations

Identifying safe and effective dosing strategies remains a challenge for both pre-clinical and clinical investigation of cannabinoids. Given the multitude of synergistic and competing mechanisms (14), proper and consistent dosing remains a major challenge. Current studies of CBD's effects on TBI suggest that CBD dosing and achieved concentrations are critical for achieving therapeutic benefits [e.g., (56)]. Other characteristics such as method of administration [e.g., (13)], duration of exposure, and temporal proximity to the TBI [e.g., (12)] further complicate efforts for achieving a clear understanding of optimal therapeutic strategies.

Beyond dosing, sex-based differences in the ECS expression, cannabinoid metabolism, and energy homeostasis regulation may impact CBD's effects, and thereby, highlight the need for further investigation into biological sex and hormone-based differences in the response to CBD (244–246). For instance, estrogen levels may affect CBD action by altering ECS-related pharmacodynamics (247) and should be considered when assessing efficacy of CBD in treating TBI (56). Furthermore, pre-clinical studies have revealed sex-dependent differences in CB_1_ receptor expression and efficacy between male and female rats: males have more, but less efficient, CB_1_ receptors than females (244, 248, 249). Since CB_1_ receptors are involved in regulating inflammation, these differences may impact the regulation of microglia in response to injury. These differences can affect the severity of the neuroinflammatory response as well as the effectiveness of CBD in treating neuroinflammation during secondary injury.

One of CBD's promising characteristics for its treatment potential is that it's generally well-tolerated (122, 250, 251). Adults can consume an acute dose of several thousand milligrams without substantial adverse effects, as demonstrated by a formal single ascending and multiple dose pharmacokinetic trial of cannabidiol oral solution. In adults, single doses of up to 6,000 mg CBD were well-tolerated, as were multiple doses of up to 1,150 mg twice daily for 6 days (252).

Clinical trials of pediatric epilepsies provide insight into the adverse effects that result from months of repeated exposure to high doses (around 10–30 mg/kg/day) (122, 253, 254). Although CBD is associated with increased risk for adverse effects such as somnolence, sedation, and impaired liver function, it's unclear if these result from CBD's interactions with other medications and therefore, it's unclear what adverse effects, if any, chronic CBD use has as a monotherapy (255). Notably, CBD's competitive inhibition of numerous cytochrome P450 liver enzymes (256) highlight the need for caution when combining CBD with additional medications that rely on metabolic breakdown by the P450 enzymes. Health-care providers, or patients concerned about discussing their off-label cannabis use with a physician, can access services such as the Cannabinoid Drug Interaction Review (CANN-DIR) (257) to identify possible interactions between CBD with concomitantly prescribed medications. Furthermore, the inconsistencies in CBD administration methods and dosing have precluded a consistent and predictable dose-response relationship that may inform TBI intervention.

### Future directions

There are currently no known blinded and randomized human clinical trials for CBD's neuroprotective effects in TBI, however, an upcoming phase II clinical trial will investigate the effects of CBD with or without Δ9-THC in patients with TBI (258). Additionally, the amalgamation of pre-clinical reports has piqued the interest among organizations with high-risk athletes. Two major sports organizations in North America whose athletes are at high risk for TBI, the National Hockey League (NHL) and the National Football League (NFL), are investigating CBD's protective and restorative effects in their athletes (259, 260). At the time of this publication, the NHL Alumni Association is moving toward a double-blind, randomized study of over 100 retired NHL players to assess the benefit of CBD on recovery from concussions (260). The NFL has also awarded funding for two clinical studies involving cannabinoids, one assessing the effects on pain and recovery from sports-related injuries and the other assessing the role of cannabinoids in pain management and neuroprotection from concussion in contact sports (259). Additionally, a separate study is recruiting participants to study CBD as a treatment for PTSD and PTSD comorbid with TBI (261), which has great relevance for those who have experienced military combat. Together, these emerging clinical trials will reveal if the promising findings from pre-clinical studies extend to at-risk human populations.

Repeated TBIs, whether from sport, combat, or other causes, increases the risk for developing chronic traumatic encephalopathy (CTE). The total number of TBIs and their severity are positively correlated with CTE risk (262), which has been neuropathologically diagnosed in 110 of the 111 NFL veterans who were tested (263). It is characterized by extensive brain atrophy, astrogliosis, myelinated axonopathy, microvascular injury, perivascular neuroinflammation, and phosphorylated tau protein pathology. The exact mechanism underlying CTE is unknown, however it emerges years after one or more TBIs. C*is* P-tau is positively correlated with axonal injury in CTE (264) and, for example, may be a targetable substrate by CBD for CTE treatment (265, 266). It is hypothesized that by mediating the effects of TBI with CBD, it may be possible to prevent the development of CTE, or at least decrease the severity of the disorder. Although more research into CTE and its underlying mechanisms is needed, the investment into clinical trials by these professional sport organizations is warranted.

## Conclusions

TBI is a public health epidemic with inconsistent clinical diagnostic criteria. Due to its complex mechanism of injury (primary and secondary) and varying severity, there is currently no single effective pharmacological treatment for TBI. CBD targets many of the cellular, molecular, and biochemical changes associated with TBI by mediating the regulation of neurotransmitters, restoring the E/I balance, preventing BBB permeability, increasing BDNF and CBF, and decreasing both ROS/NOS and microglial inflammatory responses. To accomplish this, CBD indirectly activates CB_1_R and CB_2_R while also targeting PPARγ, 5HT_1A_R, TRPV1, GPR18, and GPR55. It functions to regulate Ca^2+^ homeostasis, prevent apoptotic signaling, reduce neuroinflammation, and serve as a neuroprotectant/cerebroprotectant. *Via* a variety of targets, CBD appears to reduce cognitive (changes in memory, attention, and mood) and physiological symptoms associated with TBI, and lessen TBI-induced nociception.

There is strong mechanistic support that CBD could be an effective pharmacological intervention for TBIs, however the current state of the research field is mostly derived from rodent studies. The upcoming clinical trials will be especially informative for determining CBD's efficacy as a TBI treatment.

## Author contributions

All authors listed have made a substantial, direct, and intellectual contribution to the work and approved it for publication.
